# Treatment of Cervical Pregnancy with Ultrasound-Guided Intragestational Injection of Methotrexate: A Case Report

**DOI:** 10.1155/2021/6601461

**Published:** 2021-11-10

**Authors:** Angeliki Rouvali, Panagiotis Vlastarakos, Sofoklis Stavros, Maria Giourga, Kalliopi Pappa, Angeliki Gerede, Alexandros Rodolakis, Ekaterini Domali

**Affiliations:** ^1^1st Department of Obstetrics and Gynaecology, Alexandra Hospital, National and Kapodistrian University of Athens, Athens, Greece; ^2^Department of Obstetrics and Gynecology Democritus, University of Thrace Alexandroupolis, Greece

## Abstract

This study is aimed at describing a noninvasive conservative strategy to the treatment of cervical pregnancy and highlighting the success of ultrasound-guided therapeutic techniques. A 43-year-old woman with a history of one previous cesarean section presented in our unit with vaginal spotting and a positive urine pregnancy test. She was diagnosed with a cervical pregnancy, and she was successfully treated conservatively with the administration of intragestational sac methotrexate under ultrasound guidance. Cervical pregnancy is a rare form of ectopic pregnancy that results from conceptus implantation in the cervical canal. The main concern is the associated life-threatening hemorrhage and subsequent need for urgent hysterectomy. The evolution of ultrasound over the past decades has enabled early diagnosis and has shifted the management from a radical surgical approach towards a stepwise conservative therapeutic approach, when possible.

## 1. Introduction

A cervical ectopic pregnancy occurs when a blastocyst implant in the endocervical canal. Cervical pregnancies are relatively uncommon, and it is estimated that they account for less than one percent of all ectopic pregnancies [1]. Although this condition is rare, it has been associated with high morbidity due to the risk of major fatal hemorrhage. Early diagnosis of cervical implantation is crucial for the subsequent management that should minimize the risk of hemorrhage, eliminate the gestational tissue, and spare fertility. Nowadays, transvaginal ultrasound allows the diagnosis of cervical ectopic pregnancies at early stages, thus increasing the chances of success with medical treatment.

Although there is no consensus about the appropriate treatment of cervical pregnancies, conservative management is preferred [2, 3] in asymptomatic or minimally symptomatic and hemodynamically stable patients, leaving more radical surgical approaches such as hysterectomy for unstable patients with life-threatening symptoms and/or failure of a conservative approach. In this paper, we describe a case of cervical pregnancy which was successfully treated with ultrasound-guided intragestational sac methotrexate injection in our unit.

## 2. Case Presentation

A 43-year-old woman (gravida 2, para 1, cesarean section 1) presented in the Early Pregnancy Unit (EPU) with nausea and one-day history of painless vaginal spotting. Her last menstrual period was five weeks before admission, and she had a positive urine pregnancy test. She reported regular menstrual cycles, and she was not using any contraception. Her past medical history was unremarkable, and she had a cesarean section 13 years ago from her surgical history. She was a nonsmoker, and her body mass index was (BMI) was 20.9 kg/m^2^. Upon arrival, she was hemodynamically stable with hemoglobin of 11.2 g/dL. White blood count, platelets, liver, and renal function tests were all normal. The initial serum beta chorionic gonadotropin (*β*-hCG) level was 25.270 mIU/mL.

**Figure 1:**
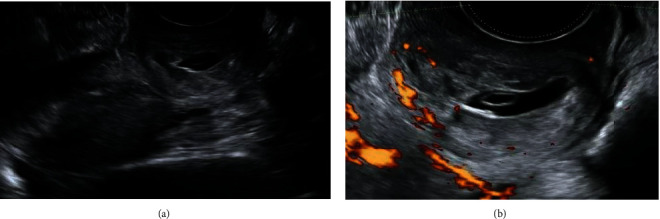
Cervical pregnancy at presentation (a) 2D transvaginal sonography in sagittal view shows an empty uterine cavity and closed internal os with ballooned cervical canal containing gestational tissue with (b) evidence of peritrophoblastic vascularity.

On physical examination, her abdomen was soft and nontender. A gentle speculum examination revealed a hyperemic cervix with gestational tissue on the anterior lip of the external cervical os and no active bleeding. Transvaginal ultrasound showed an empty uterine cavity with thick endometrial lining and a ballooned cervical canal with a gestational sac containing a yolk sac in the lower portion of the cervix and an absent “sliding sign” (Figure 1(a)). The use of Color Doppler studies showed extensive blood flow to the gestational sac (Figure 1(b)). Both ovaries were normal, and there was no evidence of free fluid. Thus, a diagnosis of cervical pregnancy was established.

**Figure 2:**
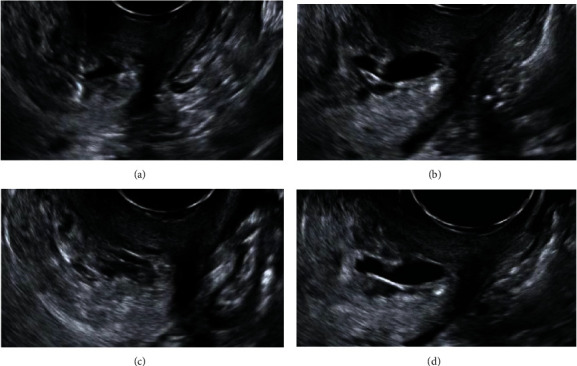
Transabdominal puncture using a 17-gauge needle under transvaginal ultrasound guidance. Aspiration of (a, b) gestational content followed by (c, d) local methotrexate administration.

Following a thorough discussion with the patient, informed consent was obtained to treat with ultrasound-guided methotrexate injection at the implantation site. Under general anesthesia, a local 75 mg dose of methotrexate was injected into the gestational sac using a 17-gauge needle (Chiba Needle, Cook Medical) under transvaginal ultrasound guidance with a prior aspiration of the gestational sac's fluid content to reduce pregnancy volume (Figure 2).

The therapeutic effect was assessed by serial monitoring of *β*-hCG levels on days one, four, and seven and transvaginal ultrasound. Posttreatment, the patient remained clinically stable, and the spotting subsided. There were no side effects associated with methotrexate administration. There was a slight decrease of *β*-hCG levels on day one and a plateau on day four, 22.605 mIU/mL and 23.136 mIU/mL, respectively. On day seven, *β*-hCG dropped to 20.075 mIU/mL and the patient was discharged. A weekly follow-up was arranged in the Early Pregnancy Unit (EPU) on an outpatient basis.

**Figure 3:**
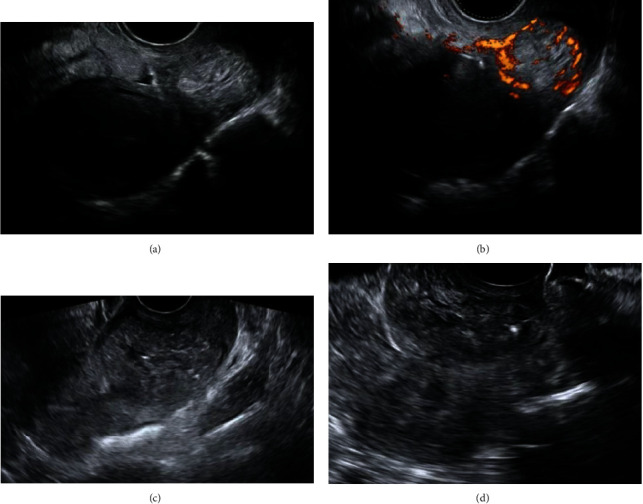
(a, b) 16 days posttreatment, *β*-hCG levels 3.689 mIU/mL, (c) 28 days posttreatment, *β*-hCG levels 305.7 mIU/mL, and (d) 31 days posttreatment, *β*-hCG levels 86.4 mIU/mL.

**Figure 4:**
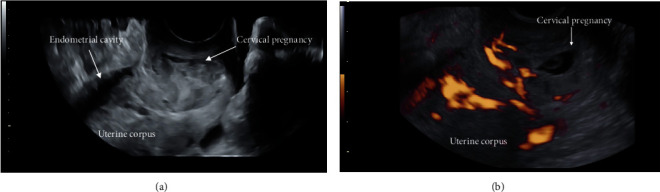
Cervical ectopic pregnancy in (a) 2D and (b) 3D transvaginal ultrasound.

During four weeks of follow-up (EPU), the patient remained asymptomatic and serum *β*-hCG levels decreased steadily from 3.689 mIU/mL on day 16 (Figures 3(a) and 3(b)) to 4 mIU/mL on day 46. Transvaginal ultrasound findings were suggestive of gestational sac regression (Figure 3).

## 3. Discussion

Cervical ectopic pregnancy is a rare entity that accounts for a small proportion of all ectopic pregnancies, and the reported incidence is approximately one per 10.000 live births [4]. It occurs when a fertilized ovum implants in the lining of the endocervix below the internal os level. Cervical pregnancy can be a life-threatening condition, and early diagnosis and treatment are essential to preserve fertility and avoid the need for hysterectomy.

The pathogenesis of ectopic pregnancy remains unclear. Prior dilatation and curettage and cesarean section have been reported as predisposing factors, possibly causing damage to the endometrial lining and the cervix [4]. Another theory suggests a rapid crossing of the fertilized ovum to the cervical canal before it is capable of implantation [5]. Cervical ectopic pregnancies have also been associated with assisted reproductive techniques [6].

Most commonly, patients with cervical ectopic pregnancy present with painless vaginal bleeding, and less than one-third of them experience lower abdominal pain [6].

Diagnosis of cervical ectopic pregnancy is based on clinical examination and ultrasound findings in a patient with a positive pregnancy test. Differential diagnosis, especially from an incomplete miscarriage or a cesarean section scar pregnancy, can be challenging. Ultrasound diagnostic criteria include (a) empty uterine cavity, (b) hourglass uterine shape with ballooned cervical canal, (c) the presence of gestational sac or placental tissue within the cervical canal, (d) absent “sliding sign,” and (e) high peritrophoblastic vascularity on Doppler examination [7].

Also, the use of three-dimensional (3D) ultrasound imaging in addition to two-dimensional (2D) scan (Figure 4) may provide additional information from the coronal section and help towards the correct diagnosis of cervical pregnancy [8, 9].

There is a wide array of therapeutic options for cervical ectopic pregnancy, varying from conservative drug therapies to radical surgical procedures. The optimal treatment method depends on the gestational age at the time of diagnosis, the clinical manifestation and its severity, the presence of coexisting viable intrauterine pregnancy, and clinician's experience.

Among the conservative management options, the use of methotrexate has revolutionized the treatment of cervical ectopic pregnancies. In cervical pregnancies, methotrexate can be administered systemically (single- or multidose regimen) with high treatment success; however, if gestational age is >9weeks, *β* − hCG levels > 10.000 IU/L, fetal cardiac activity is present, and failure rates are higher [10]. Local methotrexate injection has also been proved successful and is the proposed first-line option in cervical ectopic pregnancies with fetal cardiac activity [2]. Alternatively, potassium chloride (KCL) injection can be used as a first step [11]. Some authors suggest that curettage is necessary to reduce severe hemorrhage associated with trophoblastic shedding from the atonic cervix that occurs as a metabolic effect of methotrexate [10, 12].

Other approaches that can be used in conjunction with drug therapies or if medical treatments fail to reduce the risk of bleeding include uterine artery embolization, Foley catheter balloon tamponade [13], suction evacuation, high cervical cerclage, dilatation and curettage, and ligation of cervicovaginal branches of the uterine arteries at third and ninth o'clock positions of the cervix [14].

## 4. Conclusion

Although cervical ectopic pregnancy is a rare event, delay in diagnosis or misdiagnosis can be detrimental for the patient with life-threatening consequences. The introduction of EPUs and the widespread use of transvaginal ultrasound means that recognizing this condition is feasible early in pregnancy. Early detection enables the use of conservative therapeutic options. Intragestational injection of methotrexate with posttreatment surveillance in clinically stable patients is an effective and safe approach that preserves fertility.
